# Fibroblast Growth Factor Type 2 (FGF2) Administration Attenuated the Clinical Manifestations of Preeclampsia in a Murine Model Induced by L-NAME

**DOI:** 10.3389/fphar.2021.663044

**Published:** 2021-04-20

**Authors:** Margarita L Martinez-Fierro, Gloria Patricia Hernadez-Delgadillo, Jose Feliciano Flores-Mendoza, Claudia Daniela Alvarez-Zuñiga, Martha Lizeth Diaz-Lozano, Ivan Delgado-Enciso, Viktor Javier Romero-Diaz, Adrian Lopez-Saucedo, Iram Pablo Rodriguez-Sanchez, Ivan Alberto Marino-Martinez, Idalia Garza-Veloz

**Affiliations:** ^1^ Molecular Medicine Laboratory, Unidad Academica de Medicina Humana y C.S, Universidad Autonoma de Zacatecas, Zacatecas, Mexico; ^2^ Laboratorio de Investigacion en Farmacologia, Unidad Academica de Ciencias Quimicas, Universidad Autonoma de Zacatecas, Zacatecas, Mexico; ^3^ Department of Molecular Medicine, School of Medicine, University of Colima, Colima, Mexico; ^4^ Department of Histology, Universidad Autonoma de Nuevo Leon, Facultad de Medicina, Monterrey, Mexico; ^5^ Health Sciences Area, Universidad Autonoma de Zacatecas, Zacatecas, Mexico; ^6^ Molecular and Structural Physiology Laboratory, School of Biological Sciences, Autonomous University of Nuevo Leon, Monterrey, Mexico; ^7^ Departamento de Patologia, Facultad de Medicina, Autonomous University of Nuevo Leon, Monterrey, Mexico

**Keywords:** preeclampsia, FGF2, pregnancy hypertensive disorders, L-NAME, angiogenesis, rat model

## Abstract

**Background:** In preeclampsia, a hypertensive disorder of pregnancy, the poor remodeling of spiral arteries leads to placental hypoperfusion and ischemia, provoking generalized maternal endothelial dysfunction and, in severe cases, death. Endothelial and placental remodeling is important for correct pregnancy evolution and is mediated by cytokines and growth factors such as fibroblast growth factor type 2 (FGF2). In this study, we evaluated the effect of human recombinant FGF2 (rhFGF2) administration in a murine model of PE induced by NG-nitro-L-arginine methyl ester (L-NAME) to test if rhFGF2 administration can lessen the clinical manifestations of PE.

**Methods:** Pregnant rats were administrated with 0.9% of NaCl (vehicle), L-NAME (60 mg/kg), FGF2 (666.6 ng/kg), L-NAME+FGF2 or L-NAME + hydralazine (10 mg/kg) from the 10th to 19th days of gestation. Blood pressure (BP), urine protein concentrations and anthropometric values both rat and fetuses were assessed. Histological evaluation of organs from rats delivered by cesarean section was carried out using hematoxylin and eosin staining.

**Results:** A PE-like model was established, and it included phenotypes such as maternal hypertension, proteinuria, and fetal growth delay. Compared to the groups treated with L-NAME, the L-NAME + FGF2 group was similar to vehicle: the BP remained stable and the rats did not develop enhanced proteinuria. Both the fetuses and placentas from rats treated with L-NAME + FGF2 had similar values of weight and size compared with the vehicle.

**Conclusion:** The intravenous administration of rhFGF2 showed beneficial and hypotensive effects, reducing the clinical manifestations of PE in the evaluated model.

## Introduction

Worldwide, preeclampsia (PE), a hypertensive disorder of pregnancy, constitutes the major cause of maternal and fetal morbimortality, with an incidence of 3–10% of all pregnancies (Jeyabalan, 2013). PE is defined as new-onset hypertension (diastolic blood pressure of ≥90 mm Hg and/or systolic blood pressure of ≥140 mm Hg) and either proteinuria (≥0.3 g/day) or end-organ dysfunction after 20 weeks of gestation (WG) in a previously normotensive woman (Author Acronyms, 2013). Clinical signs appear in the second half of pregnancy, but the initial pathogenic mechanisms arise much earlier. The cytotrophoblast fails to remodel spiral arteries, leading to hypoperfusion and ischemia of the placenta that provoke generalized maternal endothelial dysfunction and consequently the clinical manifestations of disease. Until now, placenta and fetus delivery has been the only effective treatment (Martinez-Fierro et al., 2018a; Beckers and Sones, 2020).

Pregnancy is a state of maintained vasodilation and adaptations necessary to maintain vascular efficiency. In the environment of the feto-placental unit, angiogenesis is fundamental for placental development and therefore for fetal growth (Martinez-Fierro et al., 2018a; Sutton et al., 2020). During gestation, the involvement of nitric oxide (NO) is critical because it participates in several processes including implantation, uterine vascular remodeling, placental and embryonic development, and peripheral vascular resistance and vasoreactivity (Sutton et al., 2020). NO serves as the main placenta vasodilatory agent, contributing to cytotrophoblast invasion, implantation, adhesion and aggregation of intervillous platelets, and placental perfusion. Growth factors such as vascular endothelial growth factor (VEGF) and placental growth factor (PLGF), have the ability of regulate angiogenesis and to augment NO production and its modulation during pregnancy (Sutton et al., 2020). Recent evidence demonstrated that basal fibroblasts growth factor (FGF) signaling is critically required for VEGF actions that are important for both vessel formation and maintenance (Murakami et al., 2011). In experimental scenarios, FGF-driven angiogenesis is blocked by VEGF inhibition, which suggests that FGF controls angiogenesis upstream of VEGF by modulating VEGF function. FGF stimulation was shown to be necessary for the maintenance of VEGF receptor 2 (VEGFR2) levels and, in its absence, *VEGFR2* expression rapidly declined, leading to reduced production of NO, impaired angiogenesis and arteriogenesis and eventually, loss of vascular integrity (Murakami et al., 2011). FGF type 2 (FGF2 or FGFb), one of the most studied FGF family members; it induces endothelium-derived *de novo* synthesis of vasodilators, including endothelial NO, and is a very potent inducer of angiogenesis (Parsons-Wingerter et al., 2000; Murphy et al., 2001). Besides its mitogenic effect on vascular smooth muscle and endothelial cells *in vitro* and *in vivo*, previous studies suggest that FGF2 might be involved in the endothelial feedback loop activated by prohypertensive peptides and to play a role in the vascular remodeling that appears in hypertension (Ozkan et al., 2008). Although the nature of FGF-ligand interactions still remain to be elucidated during pregnancy, mesenchymal-trophoblastic and decidual-placental interactions, which are required for appropriate trophoblastic invasion, seem to be modulated by FGF and its receptors (Ozkan et al., 2008) connecting them as key participants of placental developing Hohlagschwandtner et al., 2002; Marwa et al., 2016). Under pregnancy hypertensive conditions such as PE, it has been proposed that the overload of vasopressors may be balanced by endothelial up-regulation and secretion of FGF2 as a physiologic response (Hohlagschwandtner et al., 2002). However, due to the disturbed endothelial cell function in placental tissue in severe PE cases, the regulation of this imbalance may be not possible and it may explain the lower FGF2 serum concentrations observed in women with severe PE Hohlagschwandtner et al., 2002). The relationship between the circulating FGF2 concentration and PE development has been reported in other studies, in which a decrease in FGF2 plasma concentrations was observed at 16 WG in pregnant women who subsequently developed PE when they were compared with women with normotensive pregnancies (Martinez-Fierro et al., 2015; Martinez-Fierro et al., 2018b). Based on the above, we inferred that low FGF2 circulating concentrations could be due to the poor response of the FGF2 regulatory mechanism. Therefore, by restoring its levels, the manifestations and severity of PE could be decreased or avoided. To test this hypothesis, in the present study, we generated a Sprague-Dawley rat model that simulated the gestational environment of PE. The model was induced during the rat pregnancy by administration of NG-nitro-L-arginine methyl ester (L-NAME), a known inhibitor of NO synthesis We investigated the effects of FGF2 intravenous administration on PE-related manifestations which may a possible modulator of the underlying pathologic mechanisms.

## Materials and Methods

### Animals

Forty Sprague Dawley rats (40 female and 20 male) of 8 weeks of age were obtained from the Harlan Company (Envigo, Indianapolis, IN). The female rats were kept in development and adaptation for 2 weeks until the age of 10 weeks and then were subjected to a mating protocol. Animals in the study had access to food and water ad libitum and were housed on a 12:12 h of dark-light cycle at a constant temperature of 22°C. The trial complied with the national and international legal and ethical requirements applicable to pre-clinical research. All protocols were approved by the Ethics and Biosafety Committee of the Area of Health Sciences from the Universidad Autonoma de Zacatecas (protocol ID: CEB-ACS/UAZ.Ofc.002/2015) and they were strictly carried out according with the recommendations of the *“Technical specifications for the production, care and use of laboratory animals”* Mexican guidelines (NOM-062-ZOO-1999).

### Reagents and Experimental Groups

**FIGURE 1:**
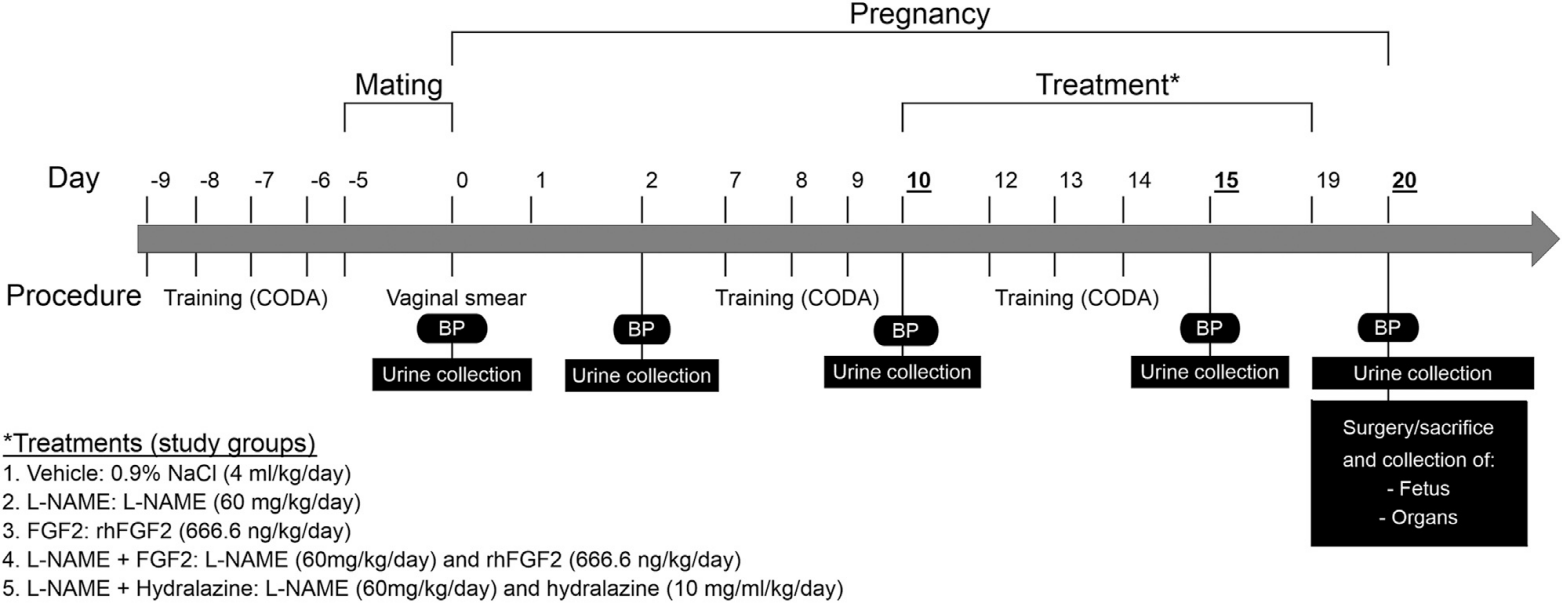
Experimental design of protocol. Schematization of the procedures, treatments and study groups included in the protocol over time. BP, Blood pressure; L-NAME, NG-nitro-L-arginine methyl ester; FGF2, fibroblast growth factor 2; rhFGF2, recombinant human FGF2.

At 10 weeks-of-age Sprague-Dawley female rats (195–210 g) were monitored for weight gain from the beginning of the mating protocol to the end of experimental protocol. Pregnancy in rats was identified by vaginal plug observation and it was confirmed by the presence of sperm in vaginal smears. Figure 1 summarizes the experimental design of protocol. Timed pregnant rats on one day of gestation were randomized to treatment groups, identified by a tattoo, and placed in their assigned treatment group (*n* = 7 per group). Five separate cohorts were required to complete the study. The experimental series consisted of the following experimental groups: vehicle, FGF2, L-NAME, L-NAME + FGF2 and L-NAME + hydralazine.

-*Vehicle group*: consisted of pregnant rats administered with saline solution (0.9% of NaCl) by intragastric route using cannula and syringe of diameter of 4 mm. The vehicle administration was done in the mornings and began on day 10 of gestation and concluded on the 19th day of pregnancy.

*-L-NAME group:* L-NAME, an inhibitor of nitric oxide synthetase, is an effective drug for the induction of hypertension in rodents (Benova et al., 2009; Possomato-Vieira et al., 2016; Amaral et al., 2018). Animals in this group were administered with L-NAME (NG-nitro-L-arginine methyl ester; Sigma-Aldrich, St Louis, MO, United States of America) by the intragastric route at a concentration of 60 mg/kg/day. The administration began on the 10th day of gestation and concluded on the 19th day of the gestation.

*-FGF-2 control group:* The FGF2 group consisted of pregnant rats treated with rhFGF2 (Sigma-Aldrich, St Louis, MO, United States of America) intravenously administered (666.6 ng/kg/day) using the tail vein (caudal). The rhFGF2 administration began on day 10 of gestation and concluded on the 19th day of pregnancy.

*-L-NAME + FGF2 group:* This group consisted of pregnant rats which were administered daily with both L-NAME (60 mg/kg/day) as mentioned before and rhFGF2 (666.6 ng/kg/day) simultaneously beginning on day 10 to 19th day of pregnancy.

-*L-NAME + hydralazine group*: the aim of this group was to compare the effect of a known antihypertensive on the maternal BP modulation and its effect at tissue level in the L-NAME model. In this group, L-NAME was administered at 60 mg/kg/day as mentioned before and oral hydralazine at 10 mg/ml/kg/day by intragastric route from the 15th to 19th day.

### Blood Pressure Determination

BP determinations in the rat-tail were carried out using a CODA® non-invasive blood pressure system (Kent Scientific Corp, Torrington, CT). Prior to BP determination, rats were trained and conditioned for enter and leave the holders for four days prior to the first BP valid measure (Figure 1). During the training window the BP determination was carried out twice a day. Rats in the protocol experienced BP measurements on days 0, 2, 10, 15 and 20, performing 25 series of measurements per rat of which 20 were taken as valid according with the manufacturer’s recommendations. Systolic blood pressure (SBP) and diastolic blood pressure (DBP) were recorded and processed using the CODA software.

### Urine Protein Determination

For the assessment of proteinuria, urine samples were collected on days 0, 2, 10, 15 and 20 of the experimental protocol (Figure 1). For this, each rat was introduced into a metabolic box and urine sample collection was carried out for 4 h. Urine samples were distributed in 50 ml tubes of volume measurement and then centrifuged at 3500 rpm for 10 min. Aliquots of 1 ml for the evaluation of protein concentration were taken from each sample and stored at −80°C. The urine protein concentration was determined using a BioRad Protein Assay (Bio-Rad, Hercules, CA) protocol in 96-well microplates. Absorbance was measured at a wavelength of 595 nm in an Appliskan^®^ microplate reader (Thermo scientific) and compared to a standard curve of known concentrations of protein. The amount of protein in the samples was determined by interpolation, reading the concentration of protein on the standard curve that corresponded to its absorbance.

### Surgical Procedures and Histological Analysis

On the 20th day of gestation, animals were anesthetized with sodium pentobarbital administered intraperitoneally a dose of 120 mg/kg. A midline laparotomy was made in each rat to expose the abdominal inferior vena cava. The inferior vena cava was cannulated with a 23-gauge needle and whole blood was collected in tubes containing EDTA. Each animal was euthanized after blood collection. Fetuses were also collected to perform measures such as weight, size and fetus width. Rat organs including heart, lung, liver, kidney, pancreas and placenta were collected and placed in 10% formaldehyde solution (pH 7) to perform the histological study. Organs in 10% neutral formaldehyde solution were fixed for 18 h, and then were dehydrated and included in paraffin. Two animals from each group were randomly selected and included in the histopathological evaluation and two cross-sectional slices were made from each organ. Tissue sections (4–6 μm thickness) were stained with hematoxylin and eosin (H&E), and periodic acid-Schiff (PAS) for traditional histopathological evaluation. For immunohistochemistry, representative sections of placenta embedded in surgipath paraplast (Leica Biosystems) were sectioned to 3 μm in thickness using a SHUR/Cut™ Microtome (Model 3500. TBS^®^, Hillsborough, NC, United States of America). Sections were deparaffinized, hydrated and blocked in 3% H_2_O_2_ solution in methanol at room temperature for 5 min. Afterward, the sections were incubated overnight at 4°C with rabbit polyclonal anti-Factor VIII primary antibody (BioCare Medical, Pacheco, CA, United States of America) diluted in 1% BSA in PBS. After three PBS washes to remove unbound primary antibody, sections were incubated with an HRP-polymer secondary antibody against rabbit IgG for 1 h and 3,3’-Diaminobenzidine (DAB) chromogen solution for 7 min at room temperature (both BioCare Medical). The slides were washed again and mounted in Fluoromount-G™ (SouthernBiotech, Birmingham, AL, United States of America), and coverslipped for microscopic observation. The slices were evaluated via images captured with an Axiocam 503 color camera, attached to an Axio Observer Z1 motorized inverted fluorescence microscope with a LD Plan-NEOFLUAR 20x/0.4 Ph2 Korr objective. The same blinded pathologist performed the analyses using ZEN Pro software (All Carl Zeiss, Jena, Germany).

### Data Analysis

Results are presented as mean ± standard error (SE) for seven animals per group. For the inferential statistics, the normal data distribution was first determined through the Kolmogorov-Smirnov test. Comparisons between two groups of data (positive control and vehicle control) were carried out by Student’s t-test or the Mann-Whitney U test. For multiple comparisons of data with a normal distribution, one-way analysis of variance (ANOVA) coupled with the Holm-Sidak method was used (BP and biochemical parameters). The data with a non-normal distribution were evaluated using the Kruskal-Wallis ANOVA on ranks and Dunn’s method as a multiple comparison procedure. One-way repeated measures ANOVA coupled with the Holm-Sidak test as post hoc test was used to evaluate if there were differences in BP values and urine protein concentrations inside the same experimental group through the evaluated times. All statistical analyses were conducted using Sigma Plot^®^ version 11 (Systat Software Inc., San Jose, CA). A 95% confidence interval (CI) was used in all the tests, and data that were different between groups at p < 0.05 were considered statistically significant.

## Results

### The Administration of L-NAME During Pregnancy in the Sprague Dawley Rats Induced PE-Like Manifestations

**FIGURE 2:**
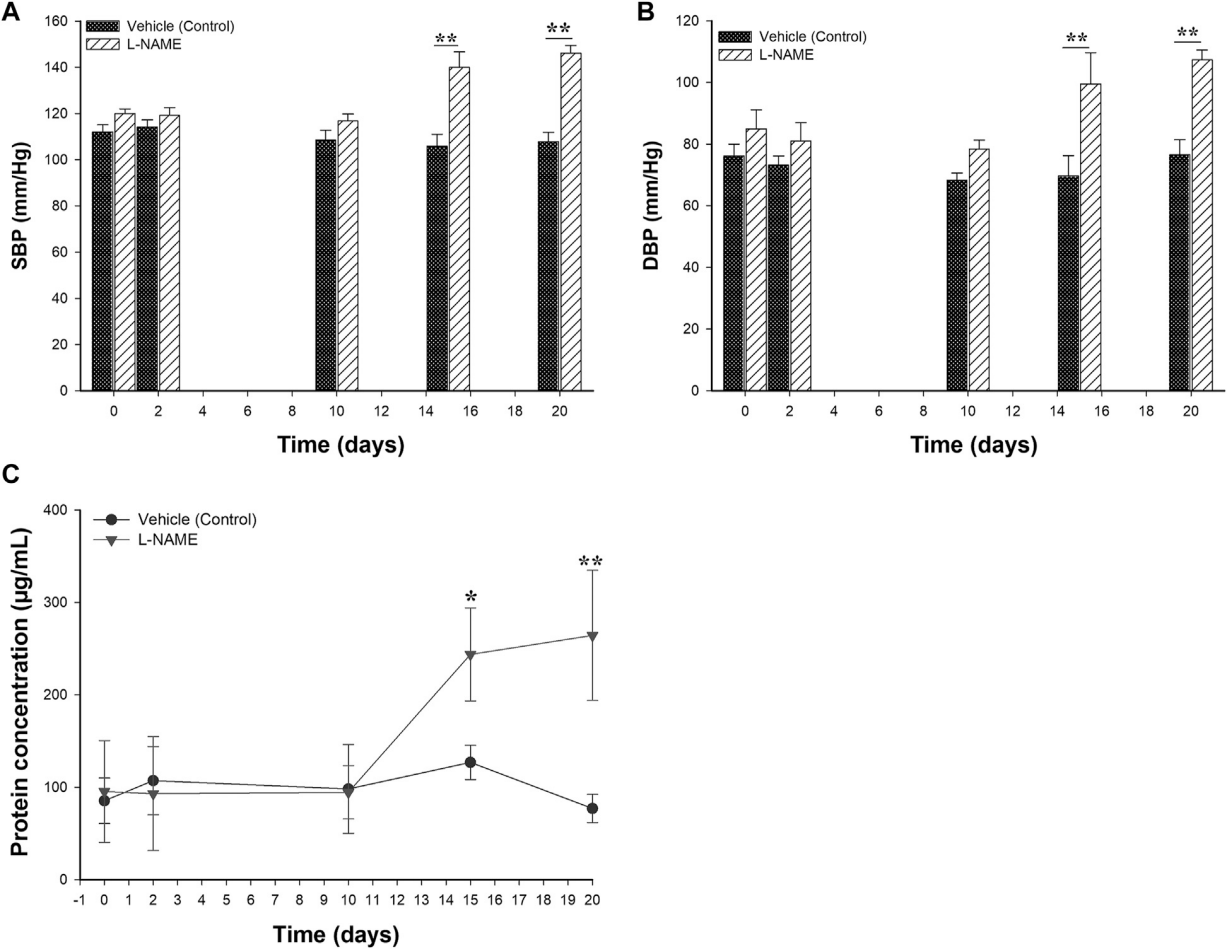
Effect of the administration of 60 mg/kg of L-NAME on blood pressure values and protein urine levels in pregnant Sprague Dawley rats. Figure 2 shows the comparison of systolic **(A)** and diastolic blood pressure **(B)** values and the urine protein concentrations **(C)** between the L-NAME and vehicle groups. Blood pressure measurements and urine protein concentrations were evaluated on days 0, 2, 10, 15 and 20 of the gestation. Data are expressed as mean ± standard error of seven rats. *p*-values obtained from the comparison between two groups were obtained using *t*-test. *p*-values obtained from comparisons inside the same group throughout the time, were calculated using repeated measures analysis of variance (ANOVA) coupled with the Holm-Sidak method. SBP: systolic blood pressure; DBP: diastolic blood pressure. *p < 0.05, **p < 0.001.

For the establishment of a PE-like model in rats, L-NAME at doses of 60 mg/kg/day or vehicle (0.9% NaCl) were administered daily to the L-NAME and vehicle groups, respectively, starting on the 10th day of gestation until the 19th day of gestation. Two clinical conditions were considered for the successful establishment of the model: hypertension (BP values ≥ 140/90) and proteinuria (urine protein concentrations >160 μg/ml). Figure 2 shows the effect of the administration of L-NAME on the SBP and DBP values. The mean of the SBP and DBP before the treatment were 118.7 mmHg ± 2.8 and 72.5 mmHg ± 3.1, in the L-NAME group and 111.6 mmHg ± 3.5 and 81.4 mmHg ± 5.0 in the vehicle group, respectively. Considering day 10 as the reference, there were differences in the BP values in the L-NAME group between days 15 and 20 (*p* < 0.05). Compared with BP values observed on the 10th day of pregnancy, in the vehicle group, there were no differences in the BP values between the time points after starting treatment (*p* > 0.05). In the comparisons of BP values between groups, there were differences both in SBP (Figure 2A) and DBP (Figure 2B) values between groups at day 15 (*p* = 0.001 for SBP and *p* = 0.023 for DBP) and at day 20 of pregnancy (*p* < 0.001 both for SBP and DBP).

To detect the presence of proteinuria, urine samples from the L-NAME and vehicle groups were taken and evaluated throughout the time using the Bradford method. Figure 2C displays the results of urine protein concentrations obtained during the experimental protocol from the L-NAME and vehicle groups. Prior to treatment, the normal urine protein concentrations ranged between 31.4 and 154.8 μg/ml. These concentrations remained constant from the 0–10 days of pregnancy and without differences in both experimental groups (*p* > 0.05). The mean of the urine protein concentrations at 10th pregnancy day (before the treatment) were 94.6 μg/ml ± 28.9 in the L-NAME group and 98.1 μg/ml ± 48.1 in the vehicle group (*p* = 0.902), respectively. There were differences in the concentration of urine protein between groups at the 15th and 20th days of pregnancy (*p* < 0.05).

According with the observed changes both in the BP and urine protein concentrations among experimental groups, the model induced by L-NAME under the experimental conditions presented in this study showed similar clinical findings to those observed in PE.

### The Administration of rhFGF2 Improved the Weight Gain During Pregnancy in the Rat PE-like Model Induced by L-NAME

**FIGURE 3:**
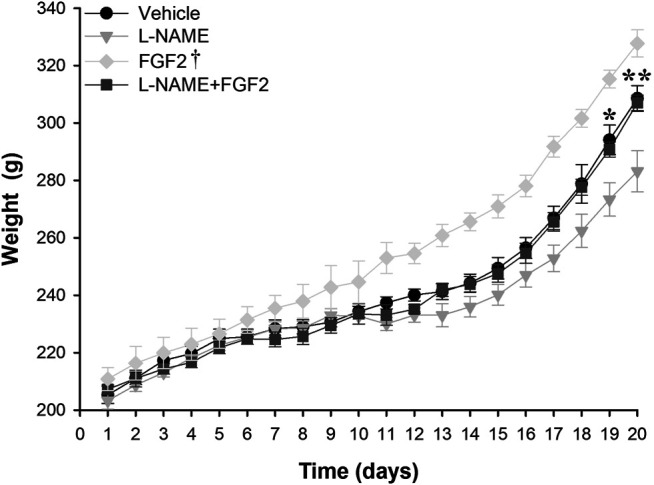
Weight gain effect of the administration of 666.6 ng/kg of rhFGF2 in Sprague Dawley rats during pregnancy in the PE-like model induced by L-NAME. The figure shows weight measurements in the vehicle, L-NAME and L-NAME + FGF2 groups during the experimental protocol. Weight measurements were taken daily. Data are expressed as mean ± standard error of seven rats. *p*-values for the comparison between groups were obtained using one-way analysis of variance (ANOVA). *p < 0.05, **p < 0.001. ^†^Compared with L-NAME, FGF2 group had *p*-values < 0.05 from day 11 to day 20.

To evaluate the effect of rhFGF2 on the weight gain/loss during the pregnancy in the PE-like model induced by L-NAME, weight gain was compared between the vehicle, L-NAME and L-NAME + FGF2 experimental groups throughout pregnancy. For this, the weight was recorded daily and plotted (Figure 3). The mean weight observed at day 10 (before starting treatment) was 233.9 g (range: 231–236.6 g). Compared with the vehicle and L-NAME + FGF2 groups, the weight gain was lower in pregnant rats treated with L-NAME alone starting at day 13 (3 days after treatment started) and was significant at day 19 of pregnancy (*p* = 0.02). At the 20th day of gestation, the mean weight observed was 308.6 ± 4.5 g, 283.6 ± 7.1 g and 307.0 ± 4.5 g, for the vehicle, L-NAME and L-NAME + FGF2 groups, respectively. Considering the L-NAME group as the reference, there was a significant difference in weight gain in the vehicle (*p* = 0.007) and FGF2 (*p* = 0.007) groups at the 20^th^ day of pregnancy. Differences in the gain/loss of weight between the vehicle and L-NAME + FGF2 groups were not observed at any time point (*p* > 0.05).

### The Administration of rhFGF2 Had a Hypotensive Effect in the Rat PE-like Model Induced by L-NAME

**FIGURE 4:**
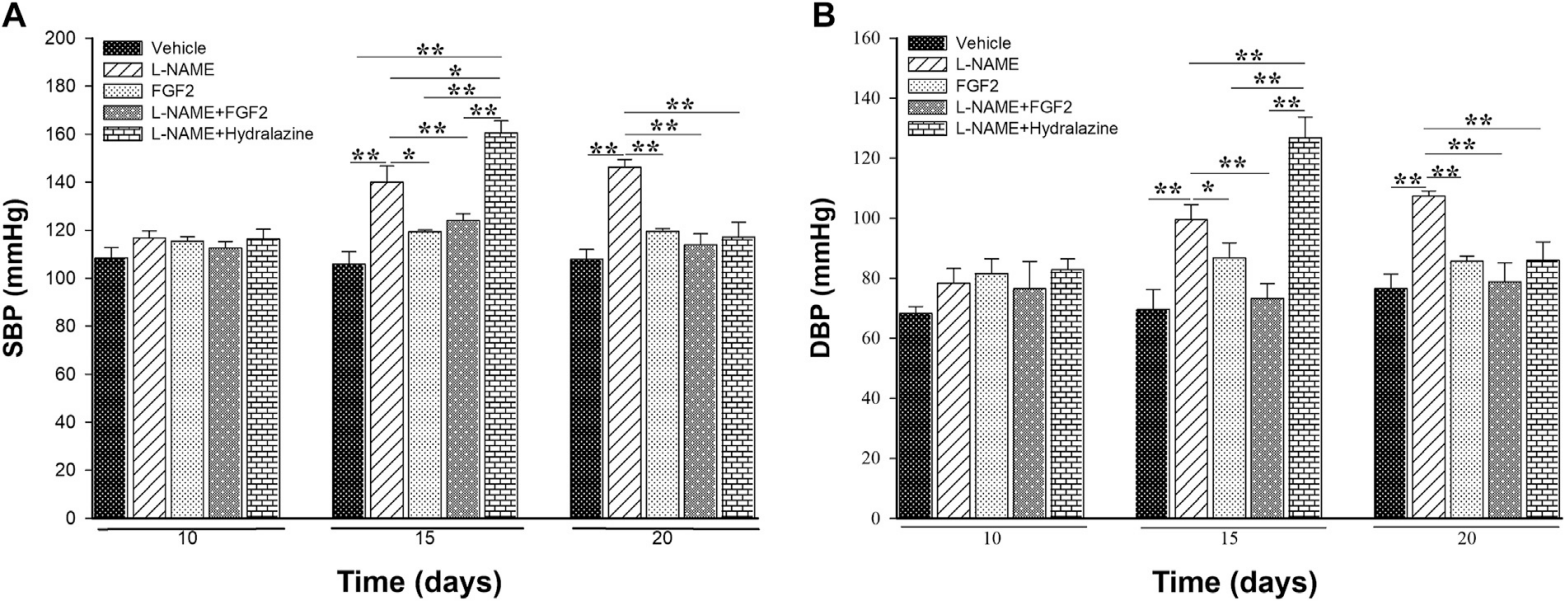
Effect of the administration of 666.6 ng/kg of rhFGF2 on the blood pressure values in pregnant Sprague Dawley rats in the PE-like model induced by L-NAME. Figure shows the measurement of systolic blood pressure **(A)** and diastolic blood pressure **(B)** among the study groups throughout the experimental protocol. Data are expressed as mean ± standard error of seven rats. *p*-values for the comparison between groups were obtained using one-way analysis of variance (ANOVA) coupled with the Holm-Sidak method. SBP: systolic blood pressure; DBP: diastolic blood pressure. *p < 0.05, **p < 0.001.

To evaluate the modulatory effect of rhFGF2 on BP values in the rat PE-like model, the vehicle, FGF2, L-NAME, L-NAME + FGF2, L-NAME + hydralazine groups were included. Figure 4 shows the results of SBP (Figure 4A) and DBP (Figure 4B) values obtained from the experimental groups for the days 10, 15 and 20. Before treatment, significant variations in BP values between the included experimental groups were not observed (*p* > 0.05). At days 15 and 20 of pregnancy and compared with the vehicle group, there were no changes in the BP values in the groups treated with rhFGF2 alone or with the group treated with L-NAME + FGF2 (*p* > 0.05). However, when L-NAME group was considered as the reference, differences in BP values with the vehicle group were observed at days 15 and 20 of pregnancy (*p* < 0.05). Differences between L-NAME *versus* L-NAME + hydralazine in SBP (*p* = 0.037) and DBP (*p* = 0.013) were observed on day 15 of pregnancy. On day 20, there was a significant increase in BP values in L-NAME group *versus* the vehicle, FGF2, L-NAME + hydralazine and L-NAME + FGF2 groups, respectively (*p* < 0.05).

In the BP multiple comparisons testing within the same group over time, as expected, in the L-NAME group there were differences both in SBP and DBP between days 10 and 15 of pregnancy (*p* < 0.05) and between days 10 and 20 (*p* < 0.001). In the same way, in the L-NAME + hydrazine group, there were differences in BP values between days 10 and 15 (*p* < 0.001) and between days 15 and 20 (*p* ≤ 0.001). There were no significant changes in BP values in the vehicle, FGF2 or L-NAME + FGF2 groups at the evaluated time points (*p* > 0.05).

### The Administration of rhFGF2 Did Not Increase the Maternal Urine Protein Concentrations But Had No Effect on the Proteinuria Observed in the PE-Like Model Induced by L-NAME

To evaluate if the rhFGF2 administration had an effect on the urine protein concentrations in the rat PE-like model induced by L-NAME, each animal from the study groups was introduced into a metabolic box for urine sample collection. Urine collection was carried out for 4 h on days 0, 2, 10, 15 and 20 of the experimental protocol. Table 1 displays the results of the urine protein concentration for each experimental group. During the gestation period, from day 0–10, there were no significant changes in urine protein concentrations between groups (*p* > 0.05). The normal urine protein concentration before treatment ranged from 31.4 µg/ml to 154.8 µg/ml. The urine protein concentrations at day 15 showed changes in the groups treated with L-NAME *vs.* vehicle (*p* < 0.001). On day 20 of pregnancy, there were differences in urine protein concentrations between the L-NAME group and the vehicle, FGF2 and L-NAME + hydralazine groups (*p* < 0.05). At this time point, the urine protein concentrations of the L-NAME + hydralazine group decreased, reaching lower levels than those obtained in the vehicle and FGF2 groups. While there were differences in urine protein concentrations between the vehicle and L-NAME + FGF2 groups (*p* < 0.001), urine protein concentrations in the FGF2 group did not differ from those observed in the vehicle group (*p* = 0.732).

**TABLE 1:** Comparison of urinary protein concentrations between study groups.

Experimental group	Urine protein concentration (µg/ml)					*p*-value^†^
	Day 0	Day 2	Day 10	Day 15	Day 20	
Vehicle	85.5 ± 24.7	107.2 ± 37.0	98.1 ± 48.1	126.1 ± 18.6	77.1 ± 15.3	0.709
FGF2	95.5 ± 29.5	97.8 ± 39.6	105.3 ± 27.0	170.9 ± 36.4	87.3 ± 12.7	0.157
L-NAME	95.4 ± 55.1	93.1 ± 61.7	94.6 ± 28.9	243.7 ± 80.3^*^	264.3 ± 70.5^*^	**0.039**
L-NAME + FGF2	114.5 ± 14.9	117.9 ± 31.7	109.3 ± 18.1	283.3 ± 32.8^*^	175.2 ± 19.2^*^	**<0.001**
L-NAME + Hydralazine	88.2 ± 26.6	92.8 ± 59.5	96.2 ± 13.2	259.0 ± 63.1^*^	41.5 ± 22.5	0.146

Protein concentration data are expressed as mean of concentration ± standard error of seven rats. ^†^ *p*-values represent the comparisons between the same group over time; in this column, significant *p*-values are highlighted in bold. For the comparisons between groups, significant *p* values (<0.05) are highlighted with one asterisk and they were calculated considering the vehicle group as the reference. *p*-values for the comparison between groups were obtained using one-way analysis of variance (ANOVA) coupled with the Holm-Sidak method.

### The rhFGF2 Administration Improved the Placental Weight Gain and Attenuated Fetal Restriction Growth in the PE-like Model Induced by L-NAME

**FIGURE 5:**
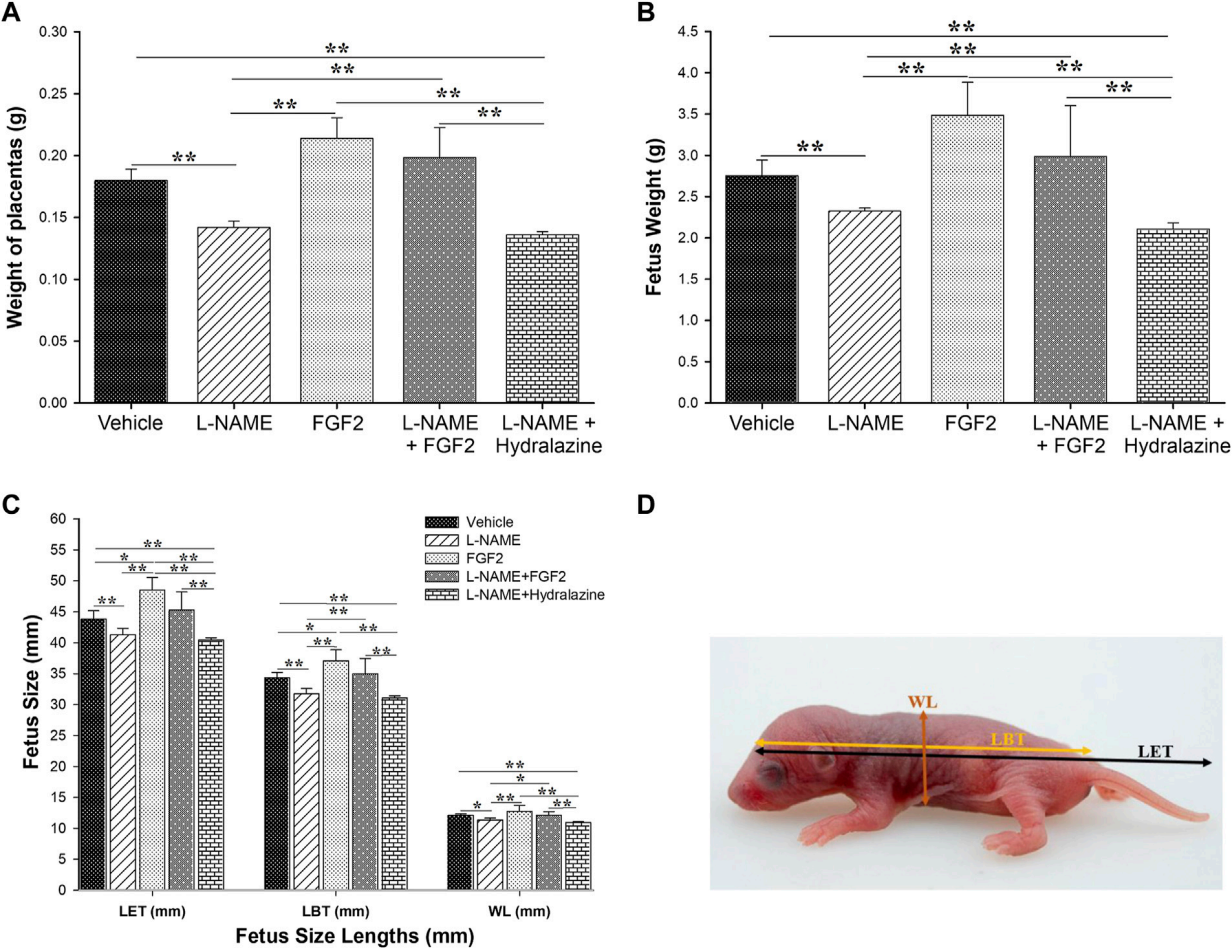
Effect of administration of 666.6 ng/kg of rhFGF2 on placental weight and fetal growth in the PE-like model induced by L-NAME. The mean of weight of the placenta and fetuses are shown in **(A,B)**. **(C,D)** shows the three different measurement of fetus (length to tail base, length to end of tail and width length) classified per experimental group. Data are expressed as mean ± standard error of seven rats. *p*-values were obtained using one way ANOVA coupled with the Dunn’s method. *p < 0.05, **p < 0.001. LBT, length to tail base; LET, length to end of tail; WT, width length.

After the experimental protocol was completed, on the 20th day of gestation, placentas and fetuses were collected to perform measures including weight, size and fetus width. The results of these measures are shown in Figure 5. The mean weight of placentas was 0.1800 g ± 0.0009, 0.2139 g ± 0.0167, 0.1420 g ± 0.0005, 0.1984 g ± 0.0242 and 0.1360 g ± 0.0002, in the of vehicle, FGF2, L-NAME, L-NAME + FGF2 and L-NAME + hydralazine groups, respectively (Figure 5A). Considering the L-NAME group as the reference, the weight of the placenta was significantly higher in the FGF2 (*p* = 0.005) and L-NAME + FGF2 (*p* = 0.001) groups. There were no differences in placenta weight between the vehicle and FGF2 groups or between the vehicle and L-NAME + FGF2 groups (*p* > 0.05). Figure 5B shows the results regarding fetus weight. The mean weight of the fetuses was higher in the vehicle (2.75 ± 0.1897 g), FGF2 (3.49 ± 0.3987 g) and L-NAME + FGF2 (2.99 ± 0.6154 g) groups when they were compared with the L-NAME group (2.32 ± 0.0388 g) (*p* < 0.05). In the evaluation of the effect of the administration of rhFGF2 on the size of the fetuses, three different measures were taken (Figures 5C,D): the length from the head to the base of the tail (LBT), the length from the head to the end of the tail (LET) and the width length (WL). The three fetal measurements were lower in the L-NAME group and in the L-NAME + hydralazine group when they were compared to the vehicle, FGF2 and L-NAME groups (*p* < 0.05). The fetal measurements of LET and LBT were higher in the FGF2 group than in the vehicle group (*p* < 0.05). There were no differences in fetal measurements between the vehicle and L-NAME + FGF2 groups (*p* > 0.05).

### Histological Effect of FGF2 Administration in the PE-like Model Induced by L-NAME

**FIGURE 6:**
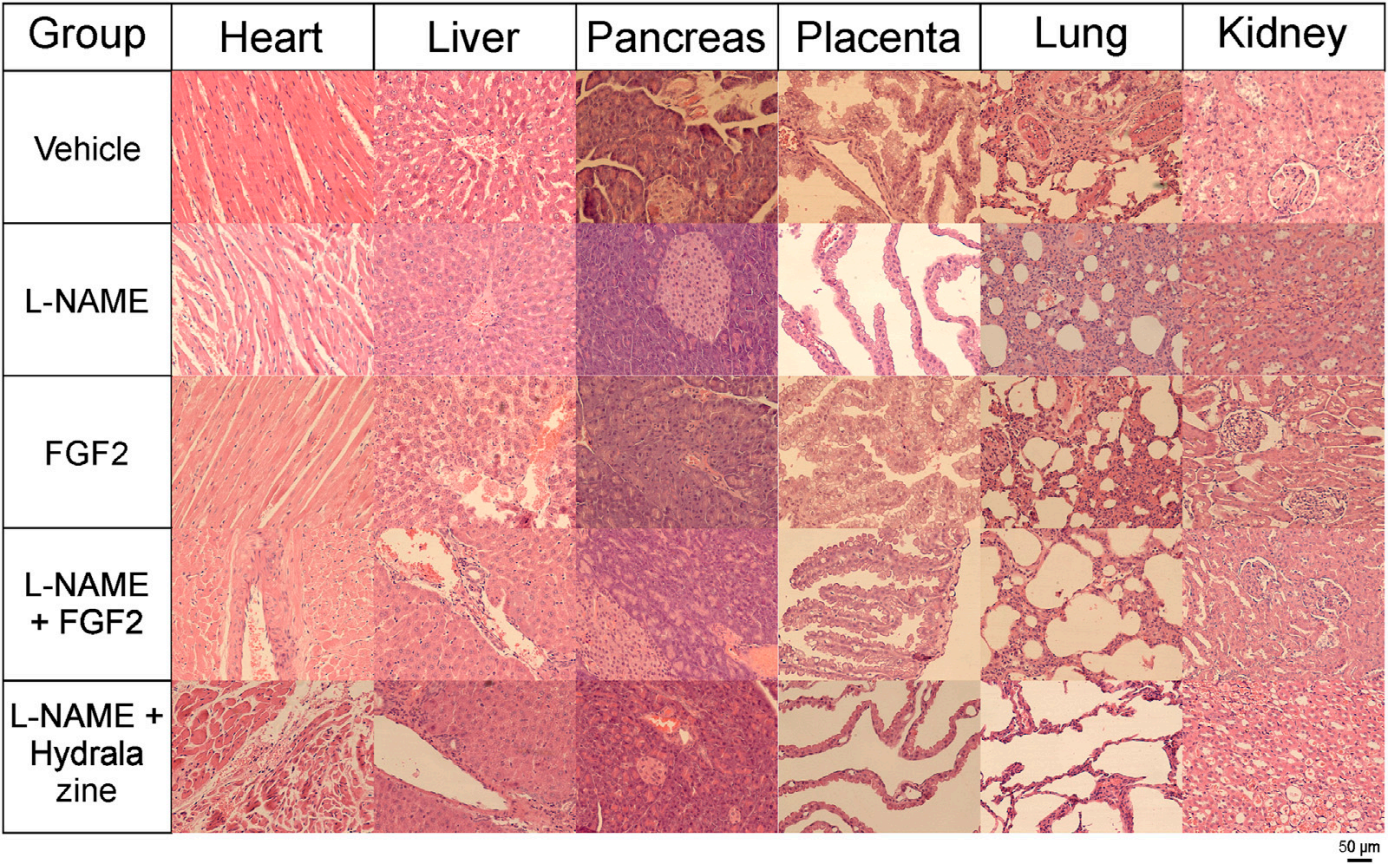
Histology of tissues from rhFGF2-treated animals (H&E staining). Tissue sections from heart, liver, pancreas, placenta, lung and kidney from all experimental groups stained with H&E and visualized with an Axiocam 503 color camera, attached to an Axio Observer Z1 motorized inverted fluorescence microscope with a LD Plan-NEOFLUAR 20x/0.4 Ph2 Korr objective.

For all the experimental groups on day 20 of pregnancy, tissue sections from the heart, liver, pancreas, lung and kidney were recovered for histological analyses. Figure 6 shows the results of the histopathological evaluation using H&E staining. The main findings for the group treated with FGF2 alone were mild congestion in the liver, congestion and focal bleeding in some regions of the placenta; in lung, there was moderate interalveolar fibrosis and foci of severe fibrotic consolidation in some areas. In the kidney, mild glomerular atrophy was observed. In the L-NAME group, the changes observed in the heart included moderate to severe vasodilation and interseptal hemorrhage; the liver showed moderate congestion with mild hepatic sinusoidal dilatation; placental vascular congestion with mild hemorrhage was observed; in the lung, there was diffuse alveolar fibrosis (by inflammatory process) and foci of severe congestion. In the kidney, there was the presence of mild glomerular atrophy and foci of inter-tubular nephritis; an angiogenic process was also documented. The L-NAME + FGF2 group showed the presence of minimal cardiac hypertrophy and slight nuclear degenerative changes and in liver, and there was congestion of the centrilobular vein and slight dilatation of the sinusoidal vein. In the pancreas, there was vascular congestion and, although the placental tissue had no histologically significant changes, a focus of edema was observed. The lung tissue had minimal focal areas of fibrosis and mild congestion of veins; slight glomerular atrophy was observed in the kidney. In the L-NAME + hydralazine group, there were mild degenerative changes in the cardiac muscle with mild cardiac damage. There was minimal vascular congestion in the liver and very minimal perivascular inflammatory infiltrates. Placental vessels were slightly dilated, but without serious histological alterations. The lung had irregular septal fibrosis and, in the kidney, there was vacuolar degradation of the distal convoluted tubules.

#### Evaluation of Angiogenesis and Patterns of Glomerular Injury

**FIGURE 7:**
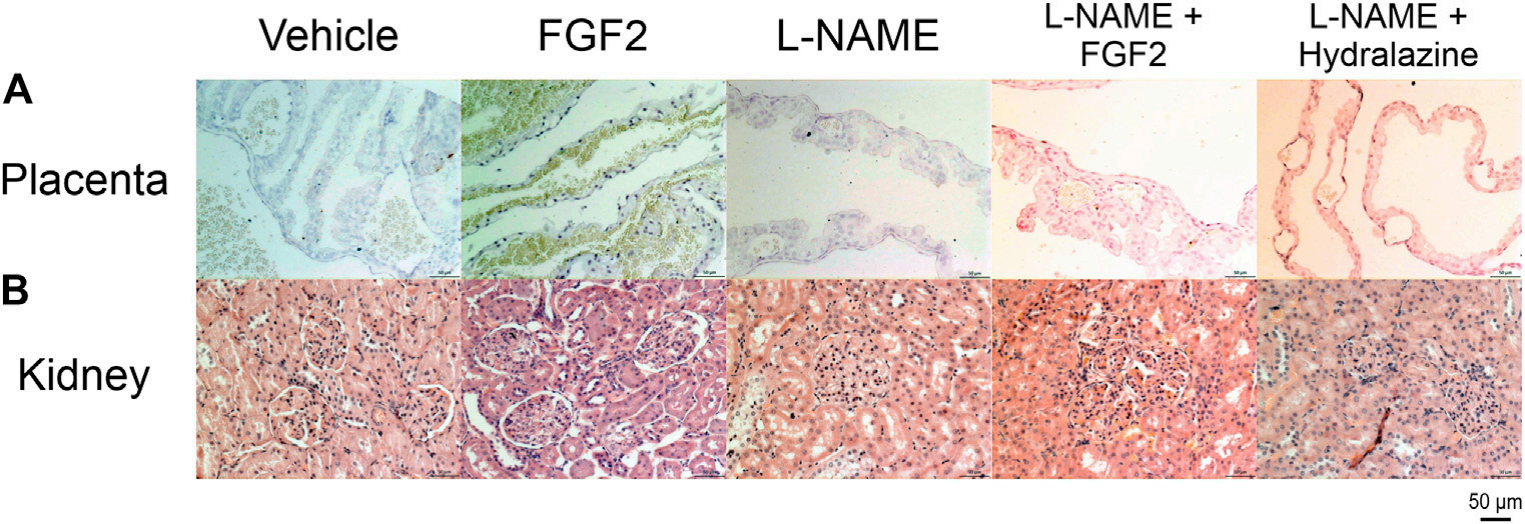
Placenta and kidney histology (immunohistochemistry against factor VIII and PAS). **(A)** Placental sections from all experimental groups immunostained for factor VIII. **(B)** kidney sections from all experimental group stained with PAS stain. All the slides were visualized at 10x and 20x with an Axiocam 503 color camera, attached to an Axio Observer Z1 motorized inverted fluorescence microscope.

In a complementary evaluation, tissue sections from the placenta and kidney were evaluated using immunocytochemistry against Factor VIII, and with PAS, respectively (Figure 7). Figure 7A shows the results of the immunocytochemistry evaluation of placenta using Factor VIII, an established marker for vascular endothelial cells, and widely used in experimental studies discovering neovascularization (Jeyabalan, 2013). The main findings for the groups of vehicle and the treated with FGF2 alone were the presence of large to medium size vessels, with a major number and homogeneously strong staining of vessels in the last group. The group of L-NAME showed small, few and faint staining vessels, while the groups of L-NAME + FGF2, and L-NAME + hydralazine showed medium size and faint staining vessels. Figure 7B shows the results of the kidney evaluation using PAS stain to discover patterns of glomerular injury. The main findings in the group treated with FGF2 were the presence of normal glomerulus along the tissue, with slight expansion of the glomerular capillary lumen. The group of L-NAME presented global severe glomerular endotheliosis, with occlusion of capillary lumens. The group of L-NAME + FGF2 showed mild glomerular endotheliosis, and the group of L-NAME + hydralazine showed mild glomerular endotheliosis with global hypercellularity that accentuates the glomerular lobulation. Angiogenesis was observed near of the renal tubules.

## Discussion

In this study, we successfully generated a Sprague Dawley rat model that simulated the clinical manifestations of PE (hypertension and proteinuria) using the NO inhibitor L-NAME at doses of 60 mg/kg/day by the intragastric route from day 10–19 of pregnancy. In this model, we evaluated if the effect of administration of rhFGF2 could lessen the clinical manifestations of PE. The role of FGF2 in PE development is not clear and there are no studies evaluating the effect of administration of this factor during human pregnancy or in an animal model of PE. However, the hypotensive effect of FGF2 administration observed in our study is in agreement with previous data indicating that both acute and chronic intravenous infusion of recombinant FGF2 in normotensive animals induces a significant reduction in BP (Dono et al., 1998; Cuevas et al., 1991). These effects have been attributed partly by the release of NO and activation of ATP-sensitive K^+^ channels (Dono et al., 1998; Cuevas et al., 1991). In a spontaneously induced hypertensive rat model, endogenous levels of FGF2 in endothelial cells were reduced and BP values were lowered by a chronic infusion of FGF2 (Dono et al., 1998; Cuevas et al., 1996). Moreover, FGF2-deficient mice were found to be hypotensive and the neural regulation of BP by the baroreceptor reflex was impaired (Dono et al., 1998). These results provide strong evidence that FGF2 participates in the vasodilator pathway, which has an important modulatory effect on BP.

Endothelial dysfunction is a key characteristic of a pregnancy complicated with PE (Martinez-Fierro et al., 2018a). The release of substances from the preeclamptic placenta and/or from damaged endothelia into the maternal circulation may act directly or indirectly upon the endothelia of end organs, including the kidney, liver and brain (Eastabrook et al., 2011; Martinez-Fierro et al., 2018a). In the glomerulus, the filtration membrane has a unique three-layer structure. Its luminal surface consists of the endothelium, the basement membrane constitutes the inner layer, and the third layer is made of podocytes with a slit diaphragm sealing the spaces between them (Kwiatkowska et al., 2020; Bertuccio, 2011). In PE, both the endothelium and podocytes are damaged, leading to proteinuria. Podocytes express all the VEGFA isoforms and they are the main sources of VEGF in the glomerulus, especially in their foot processes. VEGF produced by podocytes is moved in the opposite direction to the glomerular filtrate and has an autocrine function and paracrine effects on the endothelium (Kwiatkowska et al., 2020). Studies in which mice were deprived of VEGF produced by podocytes died at birth due to renal failure. Kidney specimens showed that the filtration membrane was abnormally developed, affecting both podocytes as well as the endothelial cells, which failed to form the fenestration typical of the glomerulus. VEGF is necessary for normal podocyte function by stimulating the phosphorylation of nephrin, which prevents podocyte apoptosis. Administration of anti-VEGF antibodies or fms-like tyrosine kinase-1 (sFlt-1), a soluble form of type 1 receptor for VEGF (VEGFR1), prevents nephrin expression in podocytes and damages them (Bertuccio, 2011). Podocytes express VEGFR1 but not VEGFR2; PE is known to be accompanied by increased levels of the sFlt-1 competing with VEGFR1 for VEGF, and this fact may explain why podocytes are more exposed to damage in PE (Kwiatkowska et al., 2020). In our results, the administration of L-NAME during rat pregnancy produced an increase in urine proteins; the administration of intravenous rhFGF2 had no effect on the proteinuria induced by L-NAME at the time points evaluated. However, there was a remarkable decrease in urine protein concentrations in the L-NAME + FGF2 group from day 15 to 20; but when compared L-NAME + FGF2 *vs* L-NAME groups, this decrement was not enough to make a statistical difference. These results can be explained by the interdependence of the endothelium and podocytes reported in other studies (Kwiatkowska et al., 2020; Baijnath et al., 2017), in which the lack of endothelial synthesis of NO causes damage to podocytes (Baijnath et al., 2017). The mechanism is not yet fully understood. However, in our study, in the kidney histological evaluation with PAS, the group of L-NAME showed global severe glomerular endotheliosis with occlusion of capillary lumens, whereas that the glomerular endotheliosis was lower in the group of L-NAME + FGF2. These results suggests that the FGF2 reduces the L-NAME associated-injuries in the glomerulus, explaining the decrement in the proteinuria observed in the L-NAME + FGF2 group from day 15–20. Additional studies will be necessary to investigate the molecular mechanism by which FGF2 decreases renal damage secondary to PE.

The kinetics of placental and fetal growth are closely interrelated. Fetal growth is dependent on nutrient availability, which in turn is related to the maternal diet, uteroplacental blood supply, placental villous development and the capacity of the villous trophoblast and fetoplacental circulation to transport these nutrients (Burton and Jauniaux, 2018). Placental-related fetal growth restriction arises primarily due to deficient remodeling of the uterine spiral arteries supplying the placenta during early pregnancy. The resultant abnormal perfusion induces cell stress within the placental tissues, leading to selective suppression of protein synthesis and reduced cell proliferation. The morphological changes are more severe in cases of growth restriction associated with PE, consistent with the greater degree of maternal vasculopathy reported (Burton and Jauniaux, 2018). In a general manner, our results show that the administration of rhFGF2 in the rat PE-like model induced by L-NAME improved the placental weight gain as well as the fetal weight and size observed in the L-NAME group, equating the measurements to that observed in the vehicle. These results are expected if we consider together all the FGF2 capacities discussed previously and that in addition to the endothelial NO induction and the interaction with the VEGF system, FGFs can also control functions of other growth factors and chemokines, such as platelet-derived growth factor (a growth factor that plays an essential role in the regulation of embryonic development, cell proliferation, cell migration, survival and chemotaxis), hepatocyte growth factor (which regulates cell growth, cell motility and morphogenesis in numerous cell and tissue types), monocyte chemoattractant protein 1 (produced by many cell types, including endothelial, smooth muscle and monocytic: regulates the migration and infiltration of monocytes, memory T lymphocytes and natural killer cells) (Deshmane et al., 2009) and angiopoietin-2 (a potent regulator of vascular branching and angiogenesis) (GeneCards, 1998; Lieu et al., 2011; Kienast et al., 2013), suggesting that FGFs can modulate multiple neovascularization events that may have an impact on placental vascular remodeling. In agreement with this hypothesis, our results showed that the L-NAME + FGF2 group had medium size blood vessels when compared with that observed with the groups of L-NAME (smaller vessels) and FGF2 (larger vessels). Together, these findings suggest that the FGF2 administration may create a more efficient placental unit and therefore have a beneficial impact on placental development and fetal growth. Additional studies are needed identify the molecular mechanism that mediate these events.

In summary, in the present study, we demonstrated that intravenous administration of rhFGF2 in a rat PE-like model induced by L-NAME improved the weight gain during pregnancy, had a hypotensive effect, did not increase maternal urine protein concentrations induced by L-NAME, improved the placental weight gain and attenuated fetal growth restriction and histologically, the morphological findings induced by L-NAME in the tissues evaluated were less severe. These results provide new insights into the role of FGF2 as a modulator of underlying pathologic mechanisms of PE in the evaluated model.

### Limitations

In our study, the administration of hydralazine started at 15th day when the hypertension has already been established. The aim of the inclusion of this experimental group was to corroborate the BP modulation in the model established and therefore the comparison of this group with the others should be considered in this context. The placental weight reported in our study represent the total weight of the collected tissue (not perfused). In spite of each plot represented the mean of seven tissues from each experimental group, these values may be higher than that observed in other studies and should be considered cautiously.

## Data Availability

The data that support the findings of this study are available from the corresponding authors, [MLMF] and [IGV] upon reasonable request.
